# Peripheral blood proteome biomarkers distinguish immunosuppressive features of cancer progression

**DOI:** 10.1002/1878-0261.13817

**Published:** 2025-02-12

**Authors:** Yeon Ji Park, Jae Won Oh, Hyewon Chung, Jung Won Kwon, Yi Rang Na, Kwang Pyo Kim, Seung Hyeok Seok

**Affiliations:** ^1^ Translational Immunology Lab, Department of Transdisciplinary Medicine Seoul National University Hospital Seoul Republic of Korea; ^2^ Cancer Research Institute Seoul National University Seoul Republic of Korea; ^3^ Department of Applied Chemistry, Institute of Natural Science Kyung Hee University Yongin Republic of Korea; ^4^ Macrophage Lab, Department of Microbiology and Immunology, and Institute of Endemic Disease Seoul National University College of Medicine Seoul Republic of Korea; ^5^ Department of Biomedical Sciences Seoul National University College of Medicine Seoul Republic of Korea; ^6^ Immunology Core Facility, Department of Translational Research Center, Biomedical Research Institute Seoul National University Hospital Seoul Republic of Korea; ^7^ Department of Biomedical Science and Technology, Kyung Hee Medical Science Research Institute Kyung Hee University Seoul Republic of Korea

**Keywords:** biomarker, immunosuppressive cancer, myeloid‐derived suppressor cells, plasma, proteomics

## Abstract

Immune status critically affects cancer progression and therapy responses. This study aimed to identify plasma proteome changes in immunosuppressive cancer and potential biomarkers predicting systemic immunosuppression. Mouse models of syngeneic breast tumors (benign 67NR and malignant 4T1) were used to collect plasma samples. Plasma samples from naive mice and both early‐ and late‐stage tumor‐bearing mice were subjected to liquid chromatography–mass spectrometry (LC–MS) analysis. 4T1‐bearing mice showed systemic immunosuppression characterized by significant generation of myeloid‐derived suppressor cells (MDSCs) as early as 7 days after tumor implantation, unlike 67NR tumors. LC–MS identified 1086 proteins across the five experimental groups, with 27 proteins showing group‐specific expression in 4T1 blood compared with 67NR blood. Immune‐related proteins osteopontin, lactotransferrin, calreticulin, and peroxiredoxin 2 were selected as potential biomarkers of MDSC‐producing breast cancer. These markers were expressed in cancer cells or MDSC in the 4T1 model, and osteopontin and peroxiredoxin 2 were associated with low survival probability and high recurrence in patients with triple‐negative breast cancer. Our findings suggest that MDSC‐producing immunosuppressive cancers have unique plasma proteomes, offering additional insights into cancer immune status.

## Graphical abstract

Immune status significantly influences cancer progression. This study used plasma proteomics to analyze benign 67NR and malignant 4T1 breast tumor models at early and late tumor stages. Immune‐related proteins–osteopontin (Spp1), lactotransferrin (Ltf), calreticulin (Calr) and peroxiredoxin 2 (Prdx2)–were associated with systemic myeloid‐derived suppressor cell (MDSC) burden, highlighting their potential roles in malignant tumors.

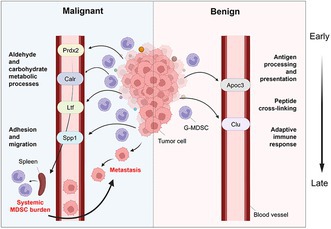

**Abbreviations**
BRCA: breast cancer
CARL: calreticulin
DFS: disease‐free survival
ELISA: enzyme‐linked immunosorbent assay
FBS: fetal bovine serum
FUSCC: Fudan University Shanghai Cancer Center
G‐MDSC: granulocytic myeloid‐derived suppressor cell
HER2: human epidermal growth factor receptor 2
LC–MS: liquid chromatography–mass spectrometry
LTF: lactotransferrin
MDSC: myeloid‐derived suppressor cell
M‐MDSC: monocytic myeloid‐derived suppressor cell
PBS: phosphate‐buffered saline
PMN: polymorphonuclear
PRDX2: peroxiredoxin 2
RFD: recurrence‐free survival
SPP1: secreted phosphoprotein 1
TCGA: The Cancer Genome Atlas
TME: tumor microenvironment
TMT: tandem mass tag
TNBC: triple‐negative breast cancer

## 1 Introduction

Cancer is a systemic disease [1]; with cancer‐related inflammation recognized as a hallmark of cancer [2, 3]. Ultimately, the global immune landscape undergoes significant alterations during tumor progression. While tumor immunology has predominantly focused on local immune responses within the tumor microenvironment (TME), it is crucial to acknowledge that immunity is coordinated across tissues. Therefore, a comprehensive understanding of immune responses to cancer must encompass both the peripheral immune system and TME.

Recent clinical and preclinical studies have revealed systemic immune perturbations that occur during tumor development, highlighting the crucial contribution of peripheral immune cells to the antitumor immune response. This disruption is prominently characterized by the expansion of immature neutrophils and monocytes, now called myeloid‐derived suppressor cells (MDSCs), in the periphery of tumor‐burdened hosts, which subsequently migrate to the TME and contribute to local immunosuppression [4, 5, 6]. Since Almand et al. described the function of increased immature myeloid cells in the peripheral blood of patients with cancer in directly inhibiting antigen‐specific T‐cell responses [7], substantial evidence now supports systemic immunosuppression. The plasma characteristics associated with cancer progression have been identified in several reports [8, 9, 10, 11]. However, changes in plasma features related to MDSC production and immunosuppressive cancer progression have not yet been studied. Blood plasma is considered a suitable matrix for proteomic analysis, offering the promise of enabling full screening and identification of cancer biomarkers. This potential holds the key to supporting cancer diagnosis and personalized medicine [12, 13]. Given the dynamic nature of plasma, which can reflect the potential pathogenic processes underlying systemic immune dysregulation during cancer progression, it would be valuable to reveal sequential plasma proteome features using immunosuppressive animal models.

We hypothesized that the plasma proteome differs between hosts bearing MDSC‐dominant tumors and those without, as MDSC generation is induced by the paracrine activity of specific cancer cells. Liquid chromatography–mass spectrometry (LC–MS) analysis of plasma from 4T1‐ and 67NR‐bearing mice, along with naive mice, revealed distinct features of the plasma proteome of MDSC‐dominant cancers. These features are expected to be associated with systemic MDSC burden and may explain the development of a suppressive immune status in patients with cancer. They also hold promise as biomarkers for diagnosing immunosuppressive MDSC‐dominant cancers.

## 2 Materials and methods

### 2.1 Animal model

All animal experiments were conducted in accordance with a study protocol approved by the Institutional Animal Care and Use Committee (IACUC) of Seoul National University (accession number SNU‐150708‐1‐2). The mice were housed in cages with constant‐flow air exchange, supporting specific pathogen‐free conditions. Seven‐week‐old female BALB/c mice were purchased from Orient Bio (Seoul, South Korea). To establish an orthotopic implantation model, 4T1 and 67NR tumor cells (10^5^ cells) were orthotopically implanted into the fourth mammary fat pad of mice. Tumor burden was evaluated by digital caliper measurement every 2–3 days following injection and calculated as tumor volume using the formula 0.5 × length × width^2^. Seven and 21 days after tumor cell injection, mice were anesthetized with ketamine/xylazine, and blood was collected through cardiac puncture using a 0.5 m EDTA‐rinsed syringe. Blood was centrifuged for 20 min at 2300 ***g***, and the plasma was isolated and stored at −80 °C until LC–MS analysis. The mammary tumor tissue, spleen, liver, and lungs were dissected and analyzed. Animal experiments were conducted in accordance with the Institute for Experimental Animals College of Medicine and the Guide for the Care and Use of Laboratory Animals prepared by the IACUC of Seoul National University.

### 2.2 Cells

Mouse breast cancer cell lines 4T1 (RRID: CVCL_0125) and 67NR (RRID:CVCL_9723) were obtained from the American Type Culture Collection. All cell lines were authenticated using short tandem repeat profiling and were tested negative for mycoplasma contamination using the Myco‐Sniff‐valid mycoplasma PCR detection kit (MPBio, Santa Ana, CA, USA). 4T1 and 67NR were maintained in RPMI1640 medium supplemented with 10% fetal bovine serum (FBS) and 1% penicillin/streptomycin (Gibco, Waltham, MA, USA). All cells were cultured at 37 °C and 5% CO_2_ in a humidified incubator.

### 2.3 Flow cytometry and cell sorting

Flow cytometry and cell sorting were performed as previously described [14]. Single cells were prepared for flow cytometry analysis. Anti‐mouse CD16/32 antibody (clone number 93) was pre‐added to block the nonspecific binding of immunoglobulins to macrophage Fc receptors. For surface staining, live cells were resuspended in staining buffers (5% FBS, 5 mm EDTA, and 1% NaN3 in phosphate‐buffered saline (PBS)) and stained with anti‐mouse CD45 (30‐F11), F4/80 (BM8), CD11b (M1/70), Gr‐1 (RB6‐8C5), Ly6G (1A8), Ly6C (HK1.4), CD8 (53.6.7), and CD3 (145‐2C11) at 4 °C for 20 min. The antibodies were obtained from eBioscience unless otherwise indicated. For intracellular staining, cells were fixed, permeabilized using 1% paraformaldehyde (Merck) and Perm/Wash Buffer (BD Biosciences, Franklin Lakes, NJ, USA), and labeled with an anti‐granzyme B antibody. Data were acquired using LSRFortessa (BD Biosciences) and analyzed using the flowjo software (Tree Star). For cell sorting, appropriately gated cell populations were sorted using a BD Aria III.

### 2.4 Cytospin and diff quick staining

Sorted MDSCs were counted and adjusted to 1.0 × 10^6^ cells·mL^−1^ in PBS. Slides were prepared using the cytocentrifugation method (cytospin) with 200 μL of input cells. The slides were fixed in 4% paraformaldehyde (Merck) for 5 min and washed twice with PBS. Diff Quick staining was performed manually. The process included initial immersion of the slides in solution no. 1 (0.1% triarylmethane), moving up and down continuously for 5–10 s. Subsequently, the extensions were immersed in solution no. 2 (0.1% xanthene) by repeating this procedure. After draining, the slides were immersed in solution no. 3 (0.1% thiazine), and the same procedure was repeated. The slides were rinsed with distilled water, air‐dried, and mounted using Permount solution (Thermo Fisher Scientific, Waltham, MA, USA). Staining solutions were purchased from Sigma‐Aldrich (St. Louis, MO, USA).

### 2.5 T‐cell proliferation assay

T‐cell proliferation assay was performed as previously described [14]. To prepare T cells, splenocytes were isolated from BALB/c mice and incubated in a 100π dish for 2 h to remove attached cells. Floating splenocytes were collected and labeled with 5 μm carboxyfluorescein succinimidyl ester (Molecular Probes) for 5 min at 37 °C. Cells were washed with complete medium twice and seeded in 96‐well round‐bottom plates precoated with αCD3 antibody (145‐2C11) (5 μg·mL^−1^) at 5 × 10^5^ cells·mL^−1^ and then incubated for 96 h. Culture supernatants were added in a 1 : 1 ratio with plain RPMI containing 10% FBS, 1% penicillin/streptomycin, 0.1% ϐ‐mercaptoethanol, and αCD28 antibody (37.51, 2 μg·mL^−1^). CD8^+^ T‐cell proliferation was examined using flow cytometry. Labeled cells were co‐cultured with sorted MDSCs (1 : 4 ratio of MDSCs to T cells).

### 2.6 Proteomic analysis

#### 2.6.1 High‐abundant protein depletion

First, 10 μL of mouse plasma was diluted up to 200 μL with Buffer A (Agilent Technology, Santa Clara, CA, USA). The diluted sample was filtered through a 0.22‐μm spin filter. The flow‐through sample was depleted using a Multiple Affinity Removal System spin cartridge following the manufacturer's protocol. The protein concentration in each sample was measured using the bicinchoninic acid assay [15].

#### 2.6.2 Filter‐aided sample preparation digestion

Filter‐aided sample preparation digestion was performed as previously described [16], with modifications. Filter‐aided sample preparation digestion was performed using 70 μg of depleted samples. Each sample was mixed with SDT lysis buffer (4% SDS, 100 mm DTT, and 100 mm Tris–HCl, pH 7.6) and incubated at room temperature for 45 min. The samples were boiled for 10 min to denature the proteins. The boiled samples were transferred to 30 kDa Microcon centrifugal filters (Millipore, Burlington, VT, USA) and centrifuged at 14 000 ***g*** for 40 min. After incubation, 45 μg of each sample was mixed with SDT lysis buffer and incubated at room temperature for 45 min. After incubation, the samples were boiled for 10 min to denature proteins. The denatured samples were then transferred to 30‐kDa Microcon centrifugal filters (Millipore) and centrifuged at 14 000 ***g*** for 40 min at 20 °C. The buffer was exchanged three times with 200 μL of 8 m urea (0.1 m Tris–HCl, pH 8.5). Alkylation of reduced cysteine bonds with 0.05 m iodoacetamide (Sigma) was performed. This was followed by three additional buffer exchanges with 8 m urea (0.1 m Tris–HCl, pH 8.5) and two buffer exchanges with 100 mm triethylammonium bicarbonate buffer, pH 8. Trypsin (Promega, Madison, WI, USA) was added to the sample at a 1:50 (w:w) enzyme‐to‐substrate ratio and incubated at 37 °C overnight. Trypsin‐digested peptides were harvested and dried using vacuum centrifugation.

#### 2.6.3 Tandem mass tag (TMT) labeling and reversed‐phase liquid fractionation at high pH

The digested peptides were labeled with the TMT 6‐plex reagent (Thermo Fisher Scientific) following the manufacturer's protocol. The digested and TMT‐labeled samples were reconstituted and loaded onto an Accucore™ 150 C18 LC Column (150 mm × 2.1 mm, 4 μm) for fractionation by an Agilent 1100 series HPLC system at a specific flow rate. The mobile phase consisted of 10 mm ammonium formate pH 10 (buffer A) and 10 mm ammonium formate and 90% acetonitrile pH 10 (buffer B). The peptides were separated using a gradient of 0–10 min, 5% B; 10–70 min, 35% B; 70–80 min, 70% B; 80–105 min, 5% B. Fractions were collected every 1 min, resulting in a total of 96 fractions, which were then combined into 12 fractions by pooling the early, mid, and late fractions. The 12 fractions were dried using vacuum centrifugation and desalted using a C18 spin column. A total of three biological replicates and one technical replicate were performed for the analysis.

#### 2.6.4 LC–MS analysis

LC–MS analysis was performed as previously described [17]. The prepared samples were resuspended in 0.1% formic acid and analyzed using a Q Exactive Orbitrap hybrid mass spectrometer (Thermo Fisher Scientific) and an Easy‐Nlc 1000 system (Thermo Fisher Scientific). The mobile phase consisted of 0.1% formic acid in high‐performance liquid chromatography water (solvent A) and 0.1% formic acid in 90% acetonitrile as solvent B. The gradient was set up linearly as follows: from 5% to 40% of solvent B for 180 min, from 40% to 80% of solvent B for 7 min, and held at 80% of solvent B for 20 min before equilibrating the column at 1% for 45 min. A 2 cm × 75 μm ID packed with a 2 μm C18 resin Trap column and a 50 cm × 75 μm ID packed with a 2 μm C18 resin analytical column were used to fractionate peptides based on their hydrophobicity. A data‐dependent acquisition method was adopted, and the top 10 precursor peaks were selected and isolated for fragmentation. The ions were scanned at high resolution, and the precursor ions were fragmented with normalized collisional energy 30. The dynamic exclusion was set at 30 s.

#### 2.6.5 Raw data processing

The acquired raw data were processed with post‐experiment monoisotopic mass refinement to increase the sensitivity of peptide identification and provide increased accuracy by selecting a unique mass class. The refined data were analyzed using Discoverer v.2.1 (Thermo Fisher Scientific). The files were searched using the Sequest HT search engine, matching the Uniprot Mouse reference (released in March 2024). Strict trypsin specificity was used, allowing for up to two missed cleavages. Carbamidomethylation (alkylation of S‐bonds in cysteine), TMT 6‐plex modification of lysine and N‐termination, and oxidation of methionine were set as the static and dynamic modifications. To ensure accurate and reliable protein identification, a stringent criterion of 1% False Discovery Rate (FDR) was applied at both the peptide and protein levels, utilizing the distribution of scores (xCorr value) corresponding to each spectrum. The quantification of each sample's reporter ion was conducted using the ‘Reporter ion Quantifier’ with TMT 6‐plex. For highly confident protein quantification, the protein ratios were calculated from at least two unique quantitative peptides in each replicate. To enrich differentially expressed proteins, the Gaussian distribution of the quantitative ratio (log_2_ value) was analyzed, and the mean and standard deviation of the Gaussian distribution were calculated. The log_2_ value ratio cutoff was set at >2 or <0.5.

#### 2.6.6 Protein selection

To identify proteins exhibiting group‐specific expression pattern statistical analyses were applied using either an ANOVA with a *P*‐value <0.005 followed by Tukey's HSD with an adjusted *P*‐value <0.05 or a Student's *t*‐test with a *P*‐value <0.05 combined with a log_2_ fold change exceeding |0.58| in comparisons between 4T1 and 67NR. Of the 27 significant proteins, acute inflammation‐associated proteins were excluded with the potential for nonspecific responses from subsequent investigations. Metabolism‐related proteins (fibrinogen‐like protein 1, alpha‐1‐antitrypsin 1–4, and triosephosphate isomerase) and cell surface proteins (CD44 antigen, complement component C9, and carcinoembryonic antigen‐related cell adhesion molecule 1) were also excluded given our focus on circulating proteins that orchestrate systemic MDSC burden.

#### 2.6.7 Functional annotation analysis and network analysis

To interpret the protein interactions among these proteins, a biological ontology search was performed. Biological processes, cellular components, and Kyoto Encyclopedia of Genes and Genomes pathways were validated using a single‐sample gene set enrichment analysis bioinformatics resource. Results with a *P*‐value <0.05 were considered significant. The entire expression matrix was used for the gene ontology search and was based on gene ontology information.

### 2.7 Immunohistochemistry

The tissues were fixed in 4% paraformaldehyde (Merck) in PBS for 2 days and sectioned at a thickness of 4.0 mm. Slides were deparaffinized and rehydrated, and antigens were retrieved in citrate buffer (10 mm sodium citrate, 0.05% Tween 20, pH 6.0) for further analysis. Primary antibodies were pre‐diluted in blocking buffer to 1:200 for osteopontin (Spp1; R&D systems, Minneapolis, MN, USA, AF‐808‐SP), lactotransferrin (Ltf; polyclonal, Merck), calreticulin (Carl; polyclonal, Thermo PA3‐900), and peroxiredoxin 2 (Prdx2; polyclonal, Merck) and were applied to tissue sections overnight at 4 °C in a humidified chamber. Biotinylated secondary antibodies were applied, followed by signal development with liquid DAB substrate (Dako). The sections were counterstained with hematoxylin (Merck).

### 2.8 Quantitative real‐time PCR (qPCR)

Real‐time PCR analysis was performed as previously described [14]. The primer sequences were as follows: mouse *Spp1* forward: 5′‐CTCCATCGTCATCATCATCG‐3′, mouse *Spp1* reverse: 5′‐TGCACCCAGATCCTATAGCC‐3′, mouse *Ltf* forward: 5′‐ACCGCAGGCTGGAACATC‐3′, mouse *Ltf* reverse: 5′‐CACCCTTCTCATCACCAATACAC‐3′, mouse *Calr* forward: 5′‐GAATACAAGGGCGAGTGGAA‐3′, mouse *Calr* reverse: 5′‐GGGGGAGTATTCAGGGTTGT‐3′, mouse *Prdx2* forward: 5′‐AACGCGCAAATCGGAAAGT‐3′, mouse *Prdx2* reverse: 5′‐AGTCCTCAGCATGGTCGCTAA‐3′.

### 2.9 Enzyme‐linked immunosorbent assay (ELISA)

Plasma levels of Spp1 (MOST00; R&D Systems), Ltf (ABIN415445, Antibodies‐online), Carl (ABIN627678, Antibodies‐online), and Prdx2 (ABIN773713, Antibodies‐online) were measured using a commercial ELISA kit, according to the manufacturer's instructions.

### 2.10 The cancer genome atlas (TCGA) analysis

#### 2.10.1 Data acquisition and preprocessing

Gene expression data were sourced from TCGA Breast Cancer (BRCA) and Fudan University Shanghai Cancer Center (FUSCC) triple‐negative breast cancer (TNBC) cohorts. Data were normalized and log_2_‐transformed for comparison across different BRCA subtypes to ensure uniformity in scale and variance.

#### 2.10.2 Survival analysis

Survival data were used to generate Kaplan–Meier plots for the basal subtype within the TCGA BRCA dataset and the human epidermal growth factor receptor 2 (HER2) subtype and for specific proteins to determine their association with patient survival outcomes. The log‐rank test was used to evaluate the statistical significance of differences in survival times between the high‐ and low‐expression groups. Kaplan–Meier survival plots were created using the ‘survminer’ package. The ‘survival’ package was used to conduct survival analyses and to calculate hazard ratios.

#### 2.10.3 Disease‐free survival (DFS) and recurrence‐free survival (RFS) analysis

DFS and RFS were analyzed to determine the prognostic value of protein expression in BRCA subtypes. Survival curves were plotted, and log‐rank *P*‐values were computed to discern the differences in DFS between patients with high and low expression of the proteins of interest. RFS analysis was specifically performed on the FUSCC TNBC cohort, comparing the protein expression in patients with no recurrence versus those with recurrence.

### 2.11 Quantification and statistical analysis

GraphPad Prism 6 (GraphPad Software) was used for statistical analysis. Results are presented as means ± SEM for at least three independent experiments unless otherwise indicated. Unpaired two‐tailed Student's *t*‐tests were used to compare two groups of independent samples, and one‐way or two‐way analysis of variance (ANOVA) with Tukey's *post hoc* test was used to compare multiple groups. Sample sizes (*n*) are indicated in the figure legends. Results with *P*‐values of <0.05 were considered to be statistically significant.

## 3 Results

### 3.1 4T1 BRCA model demonstrates a systemic MDSC burden

**Fig. 1:**
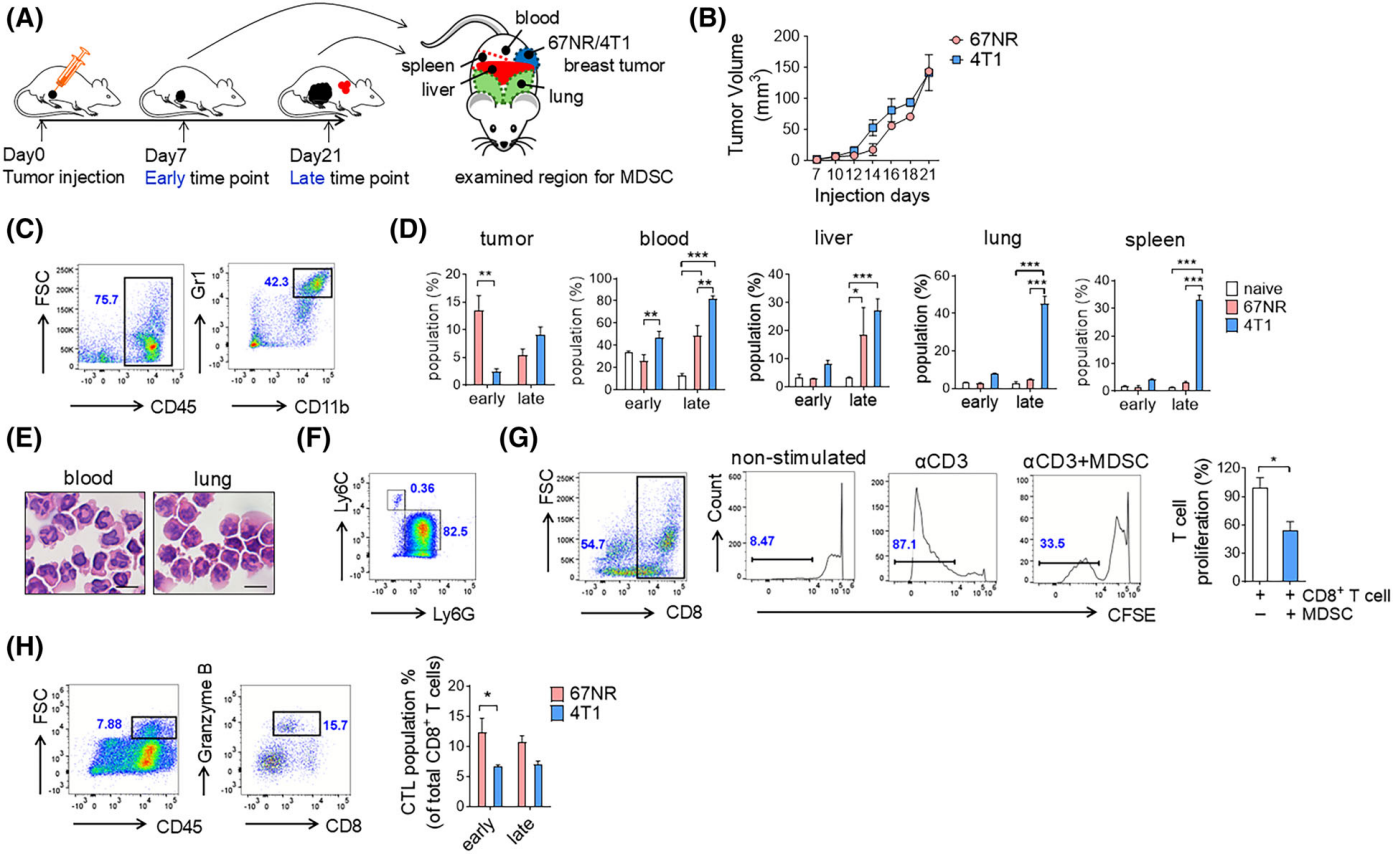
Systemic immunosuppressive myeloid‐derived suppressor cell (MDSC) burden in 4T1‐bearing mice. (A) Schematic illustration of the experimental design. Tumor cells (67NR or 4T1) were orthotopically injected into female BALB/c mice on day 0. After seven and 21 days, breast tumors, blood, liver, lung, and spleen were collected. (B) Tumor growth analysis of 67NR‐bearing and 4T1‐bearing mice, and there were no significant differences in tumor volume between the two models (*n* = 2). (C) Gating strategy for MDSCs (CD45^+^ CD11b^+^ Gr1^+^) (*n* = 4). (D) Proportions of MDSCs in tumors, blood, liver, lung, and spleen in naive, 67NR‐bearing, and 4T1‐bearing mice (naive early *n* = 3; naive late *n* = 3; 67NR early *n* = 4; 67NR late *n* = 2; 4T1 early *n* = 4; 4T1 late *n* = 5). Statistical testing was done by two‐way ANOVA (mean ± SEM, *P < 0.05, **P < 0.01, ***P < 0.001). (E) Diff Quik staining demonstrated that sorted MDSCs from blood and lung samples of 4T1‐bearing mice were G‐MDSCs (*n* = 2). Scale bar = 20 μm. (F) Double dot plot showing Ly6G and Ly6C expression within CD11b^+^ Gr1^+^ cells as analyzed in (c). (G) Carboxyfluorescein succinimidyl ester (CFSE) dilution assays in non‐stimulated T cells (*n* = 2), T cells stimulated by anti‐CD3 antibody (aCD3, *n* = 6), and T cells stimulated by aCD3 and co‐cultured with MDSCs (aCD3 + MDSC, *n* = 3) demonstrated the immunosuppressive function of MDSCs. Values in brackets indicate the percentage of CFSE‐negative cells among the total CD8^+^ T cell population. Statistical testing was done by unpaired two‐tailed Student's t‐test (mean ± SEM, *P < 0.05). (H) Flow cytometry analysis showed the numbers of granzyme B^+^ cytotoxic T‐cell populations of total CD8^+^ T cells in 67NR and 4T1 tumors (*n* = 3). Statistical testing was done by two‐way ANOVA (mean ± SEM, *P < 0.05).

Murine 4T1 cells, originally isolated from a spontaneous mammary tumor in BALB/c mice, are metastatic and exhibit characteristics of the human basal/TNBC subtype [18]. In contrast, 67NR murine cell lines have been shown to be less invasive [19, 20]. The 4T1 model has also been shown to induce the expansion of MDSCs, which regulates the metastatic cascade [21]. To confirm the immunosuppressive activity of MDSCs [22], we orthotopically injected 67NR and 4T1 tumor cells into BALB/c mice (Fig. 1a). Tumor growth rates were not significantly different between the two models, although 4T1‐bearing mice eventually died of lung metastasis (Fig. 1b). To address temporal MDSC production, we isolated blood, spleen, liver, and lung, as well as tumor tissue, at early (7 days) and late (21 days) time points after injection and analyzed the frequency of CD11b^+^ Gr1^+^ cells (Fig. 1c) during the course of the disease. At an early time point, 67NR‐bearing mice exhibited a higher MDSC population in tumors than 4T1‐bearing mice. However, 4T1‐bearing mice showed higher MDSC numbers in the blood at both time points and in the liver at the late time point than 67NR‐bearing mice. The MDSC population in the lungs and spleen was significantly higher at the late time point but not in the tumor (Fig. 1d).

MDSCs are categorized into two main subsets based on Ly6G and Ly6C expression. Granulocytic MDSCs (G‐MDSCs) are Ly6G^high^ Ly6C^low^, and monocytic MDSCs (M‐MDSCs) are Ly6G^−^ Ly6C^high^ [23]. To confirm that the CD11b^+^ Gr1^+^ population represented MDSCs, we performed Diff Quik staining of cells sorted from the blood and lungs of 4T1‐bearing mice (Fig. 1e). Morphological examination of these cells supported their identity as G‐MDSCs characterized by a horseshoe‐shaped nucleus and granules inside the cells. We further demonstrated a predominant population of Ly6G^high^ Ly6C^low^ cells within the CD11b^+^ Gr1^+^ cell population using flow cytometry (Fig. 1f). Additionally, we confirmed their direct immunosuppressive function by performing a T‐cell proliferation assay (Fig. 1g). MDSCs isolated from 4T1 tumors significantly inhibited CD8^+^ T‐cell proliferation. Consistent with this result, the population of granzyme B‐expressing cytotoxic T cells was lower in 4T1‐bearing mice (Fig. 1h). These results demonstrate that systemic MDSC burden is detected even in the relatively early stages of 4T1 cancer progression and has the ability to suppress CD8 T‐cell activity.

### 3.2 Proteomic analysis on plasma identified unique protein changes in the blood of 4T1‐bearing mice

**Fig. 2:**
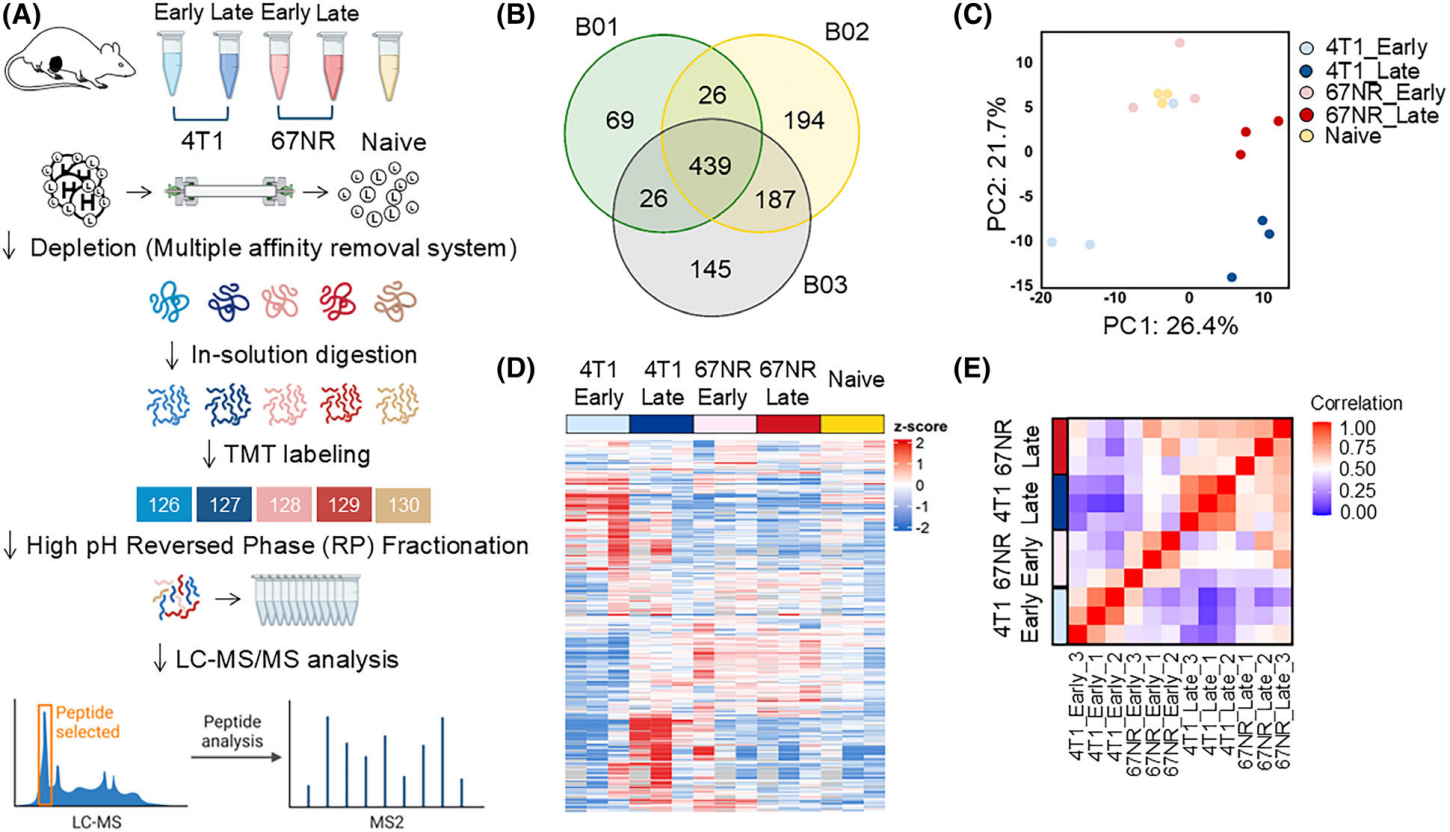
Proteomic workflow for uncovering alterations in plasma proteome of MDSC‐dominant breast cancer. (A) Workflow diagram showing the experimental design and proteomic analysis process. (B) Venn diagram representing the overlap of identified proteins across three batches (B01, B02, and B03), showing shared and unique protein identifications. (C) Principal component analysis (PCA) plot demonstrating the separation of proteomic profiles for early‐ and late‐stage of 4T1 and 67NR models, including naive controls. (D) Heatmap of differentially expressed proteins, with rows representing individual proteins and columns representing sample groups. (E) Correlation heatmap showing the pairwise comparisons between the sample groups.

Next, we performed quantitative proteomics on mouse plasma samples from the five groups (67NR early, 67NR late, 4T1 early, 4T1 late, and naive) with three biological replicates (Fig. 2a). High‐abundance proteins were depleted to remove albumin, transferrin, and immunoglobulins, which interfere with the detection of low‐abundance proteins using liquid chromatography–tandem mass spectrometry. The depleted samples were digested and TMT labeled for relative quantification. Each TMT chemical was labeled for each group, and the labeled samples were pooled and fractionated to boost proteome identification. Desalted peptides were injected into a high‐resolution, high‐scan‐speed mass spectrometer coupled with a nano‐LC. A total of 1086 plasma proteins were identified with more than one unique and razor peptide per protein, applying a stringent 1% FDR at both the peptide and protein levels from triple biological replications, and 439 proteins were overlapped through three biological replicates (Fig. 2b). Each identified protein was composed of more than one unique and razor peptide (Table S1), and we selected the proteins with more than two unique and razor peptides and performed statistical analysis (Table S2).

Through the implementation of principal component analysis, hierarchical clustering, and correlation analyses as basic quality checks, it was determined that the late stages of 4T1 and 67NR exhibited more significant differences than the early and naive‐states (Fig. 2c–e). These differences between the late stages of 4T1 and 67NR can be interpreted as variations in tumor progression. Additionally, the early 4T1 stage was found to have more pronounced differences in correlation than the other groups. These findings suggest that the progression of malignant cancer is intricately linked to specific protein patterns in the blood, underscoring the potential of these findings for understanding cancer dynamics.

### 3.3 MDSC‐dominant cancer progression is characterized by distinctive plasma proteins

**Fig. 3:**
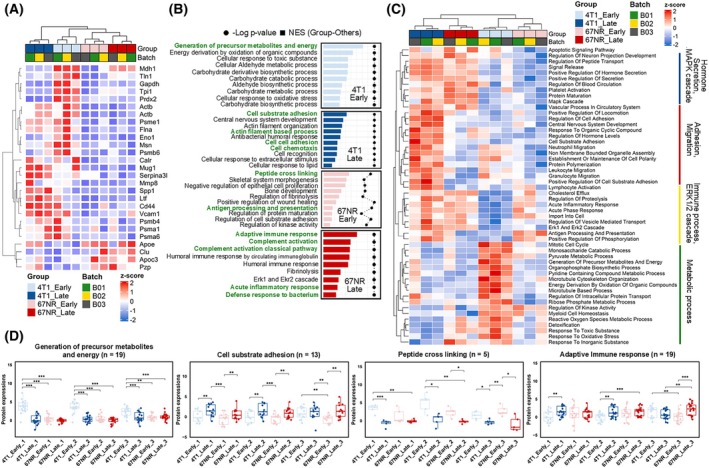
Gene Ontology and heat map showing upregulated genes and proteins in tumor‐bearing mice. (A) Heat map displaying the z‐score normalized expression levels of proteins across groups and batches. With a strict cutoff from statistical analysis, 27 significant proteins are selected. (B) Bar plot of −log *P*‐value against normalized enrichment score (NES) for biological processes identified as significant, with the size of the circle representing the *P*‐value and the color indicating the NES. Biological processes discussed in the main text are highlighted in green. (C) Clustered heatmap of biological processes with annotations, showing differences in protein expression levels across the sample groups and functional processes. (D) Box plots for selected biological processes, highlighting significant proteins with differential expression between 4T1 and 67NR models at different stages. Comparisons were performed using Student's *t*‐test (error bars represent 25–75 percentiles, *P < 0.05, **P < 0.01, ***P < 0.001).

To identify the proteins linked to the generation of MDSCs and their systemic impact, we conducted an analysis based on the intensity of quantifiable proteins. The intensities were normalized to the naive intensity for subsequent statistical evaluations. A total of 27 proteins demonstrating distinct group‐specific expression patterns were identified. These proteins were selected based on statistical significance derived from either an ANOVA with a *P*‐value <0.005 followed by Tukey's HSD with an adjusted *P*‐value <0.05 or a Student's *t*‐test with a *P*‐value <0.05 combined with a log2 fold change exceeding |0.58| in comparisons between 4T1 and 67NR. Furthermore, these proteins, characterized by a high interaction score with a ‘Core score’ >0.5, indicate their crucial roles within the protein interaction network, suggesting they are core proteins in the system. This enrichment of specific proteins highlights their potential importance in understanding the differences between 4T1 and 67NR blood samples (Fig. 3a).

Among the core proteins identified, a significant number increased in the late and earlystages of 4T1, including Vcam1, Mmp8, Psma6, Eno1, Msn, Actb, Psme1, and Flna. Immune cell adhesion molecules (Actb, Vcam1, Flna, and Cd44) and immune‐related factors (Calr, Spp1, and Ltf) increased during the late 4T1 stage (Fig. 3b). In the early stage of 4T1, proteins associated with aldehyde and carbohydrate metabolic processes (Tpi1, Gapdh, Pgk1, and Eno1) and antioxidants (Cat, Prdx2) were significantly elevated. The adaptive immune response was strongly enriched in the late stage of 67NR but not in 4T1, implying the predictability of plasma proteome data in discriminating the systemic immune status.

When analyzing stage‐specific processes using the normalized enrichment score, the earlystage of 4T1 showed a significant upregulation in pathways related to the generation of precursor metabolites and energy, which are central to metabolic processes (Fig. 3c). In contrast, the late stage of 4T1 displayed an increase in adhesion‐ and chemotaxis‐related processes. The early stage of 67NR was characterized by the enhancement of pathways such as peptide cross‐linking, wound healing, antigen processing, and presentation. A commonality observed in the late stages was the increase in hormone secretion and EGFR‐centered Erk1, Erk2 cascade, and MAPK cascade, whereas no specific commonalities were identified in the early stages (Fig. 3d).

Of the 27 significant proteins, we excluded acute inflammation‐associated proteins, metabolism‐related proteins (fibrinogen‐like protein 1, alpha‐1‐antitrypsin 1–4, and triosephosphate isomerase), and cell surface proteins (CD44 antigen, complement component C9, and carcinoembryonic antigen‐related cell adhesion molecule 1), based on the criteria described in the methods section. Subsequently, a selection process led to the identification of proteins, such as Spp1, Ltf, and Calr, all of which were aligned with the immune response and observed exclusively in the advanced stages of 4T1 progression. Additionally, Prdx2, exclusively upregulated during the early stages of 4T1 progression, was incorporated for further analysis.

### 3.4 Four proteins were validated to be present in the plasma of 4T1‐bearing mice

**Fig. 4:**
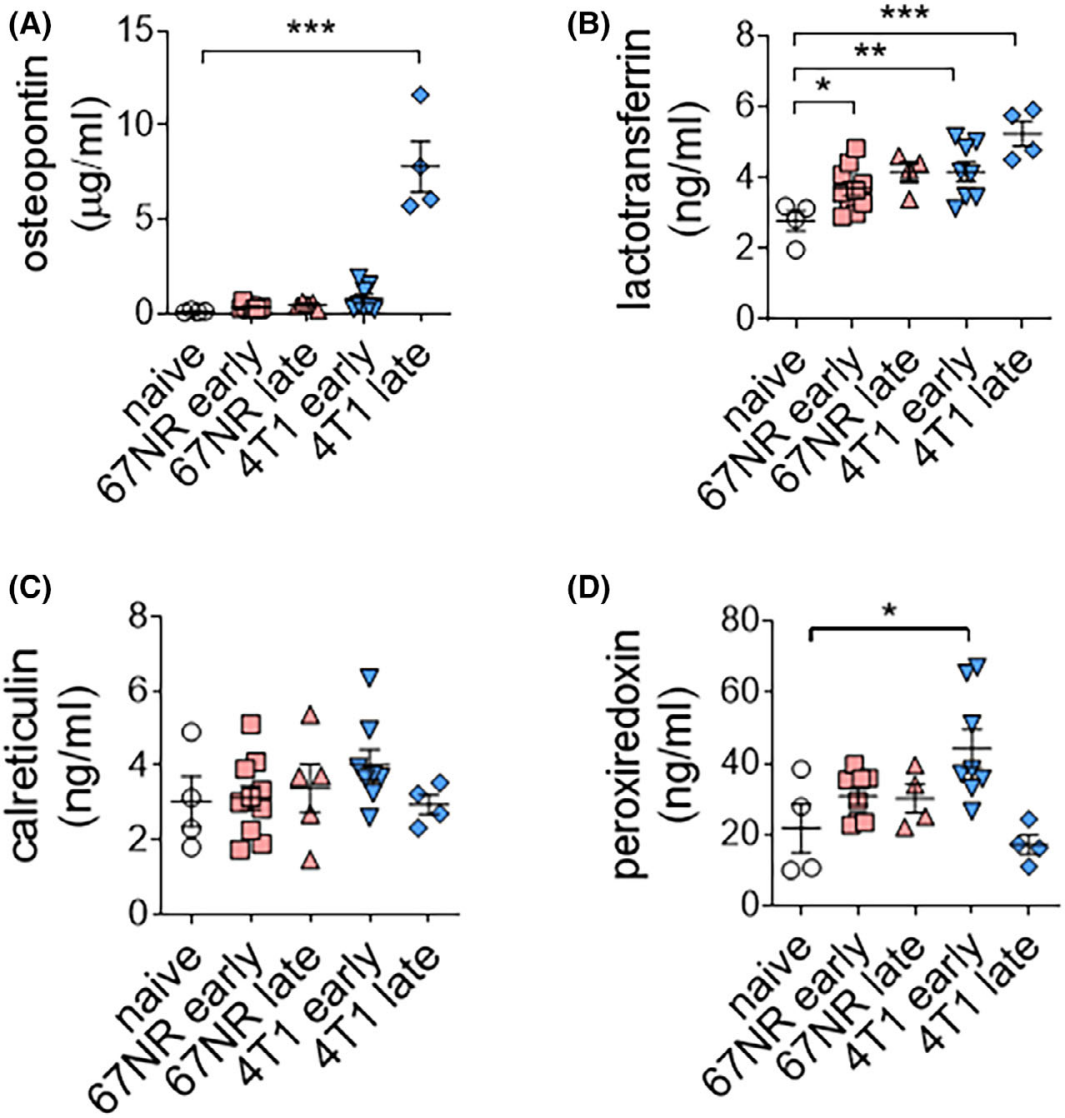
Potential biomarker levels in plasma. (A) Osteopontin (Spp1) levels in different groups (naive *n* = 4; 67NR early *n* = 10; 67NR late *n* = 5; 4T1 early *n* = 8; 4T1 late *n* = 4). (B) Lactotransferrin (Ltf) levels in different groups (naive *n* = 4; 67NR early *n* = 10; 67NR late *n* = 4; 4T1 early *n* = 8; 4T1 late *n* = 4). (C) Calreticulin (Calr) levels in different groups (naive *n* = 4; 67NR early *n* = 10; 67NR late *n* = 5; 4T1 early *n* = 8; 4T1 late *n* = 4). (D) Peroxiredoxin 2 (Prdx2) levels in different groups (naive *n* = 4; 67NR early *n* = 8; 67NR late *n* = 4; 4T1 early *n* = 8; 4T1 late *n* = 4). Statistical testing was done by one‐way ANOVA (mean ± SEM, *P < 0.05, **P < 0.01, ***P < 0.001).

To validate the results of the plasma mass data, we conducted ELISA for four proteins–Spp1, Ltf, Calr, and Prdx2–in the plasma of 67NR and 4T1‐bearing mice. The level of Spp1 was significantly increased only at a late time point in 4T1‐bearing mice (Fig. 4a). Ltf was highly detected in the plasma of 67NR‐bearing mice; however, this increase was not as significant as that in 4T1‐bearing mice (Fig. 4b). Calr levels did not differ between 67NR and 4T1‐bearing mice (Fig. 4c). Prdx2, which specifically increased in the early phase of 4T1, was elevated only at an early time point in 4T1‐bearing mice (Fig. 4d). Three of the four selected biomarkers were validated in plasma.

### 3.5 Enriched biomarkers have different cellular origins

**Fig. 5:**
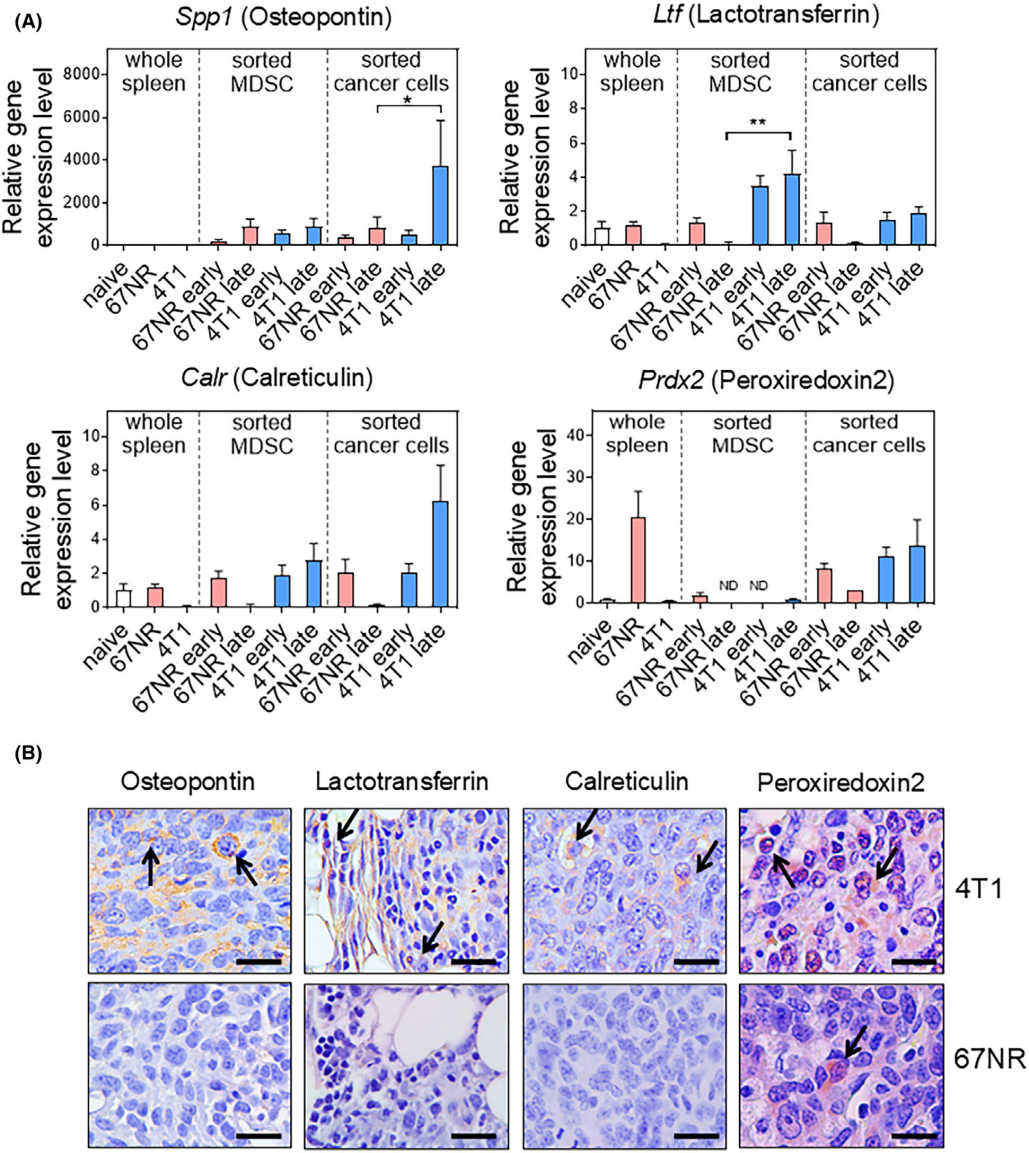
Confirmation of major cell populations producing candidate biomarkers. (A) Relative mRNA expression levels of *Spp1, Ltf, Calr*, and *Prdx2* at the whole spleen, sorted MDSCs, and cancer cells from 67NR‐bearing, 4T1‐bearing, and naive mice (naive spleen *n* = 4; 67NR spleen *n* = 8, 4T1 spleen *n* = 8; 67NR early MDSC *n* = 8; 67NR late MDSC *n* = 5; 4T1 early MDSC *n* = 7; 4T1 late MDSC *n* = 8; 67NR early cancer cell *n* = 8; 67NR late cancer cell *n* = 4; 4T1 early cancer cell *n* = 9; 4T1 late cancer cell *n* = 9). Statistical testing was done by one‐way ANOVA (mean ± SEM, *P < 0.05, **P < 0.01). (B) Immunohistochemistry (IHC) showed the expression of Spp1, Ltf, Calr, and Prdx2 in 4T1 and 67NR tumor samples (*n* = 2). Black arrows indicate protein expression in tumor cells and stromal cells. Scale bar = 50 μm.

We hypothesized that the enriched biomarkers were expressed in cancer and immune cells, including MDSCs. To determine the primary cell populations expressing *Spp1* (Spp1), *Ltf* (Ltf), *Calr* (Calr), and *Prdx2* (Prdx2), we conducted qPCR analysis of whole spleen tissue, where MDSCs were mainly generated, MDSCs sorted from tumors, and cancer cells (Fig. 5a). *Spp1* gene expression was the highest in 4T1 cancer cells at the late time point, whereas *Ltf* gene expression was the highest in MDSCs of 4T1‐bearing mice at the late time point. Similar to *Spp1*, *Calr* expression was the most prominent in late‐stage 4T1 cancer cells, although the difference was not statistically significant. The spleens of 67NR‐bearing mice showed the highest expression level of *Prdx2*, and the cancer cells of 4T1 at both early and late stages expressed similar levels of *Prdx2*. Next, we conducted immunohistochemistry analysis of tumor samples from 67NR and 4T1 models to identify the cellular origin of candidate protein expression (Fig. 5b). Spp1, Calr, and Prdx2 were highly expressed in 4T1 cancer cells, whereas Ltf was highly expressed in MDSC within 4T1 tumors. Our findings suggest that MDSCs or cancer cells in 4T1 tumors express Spp1, Ltf, Calr, and Prdx2, potentially influencing elevated plasma concentrations.

### 3.6 Identified potential biomarkers have clinical relevance in patients with BRCA

**Fig. 6:**
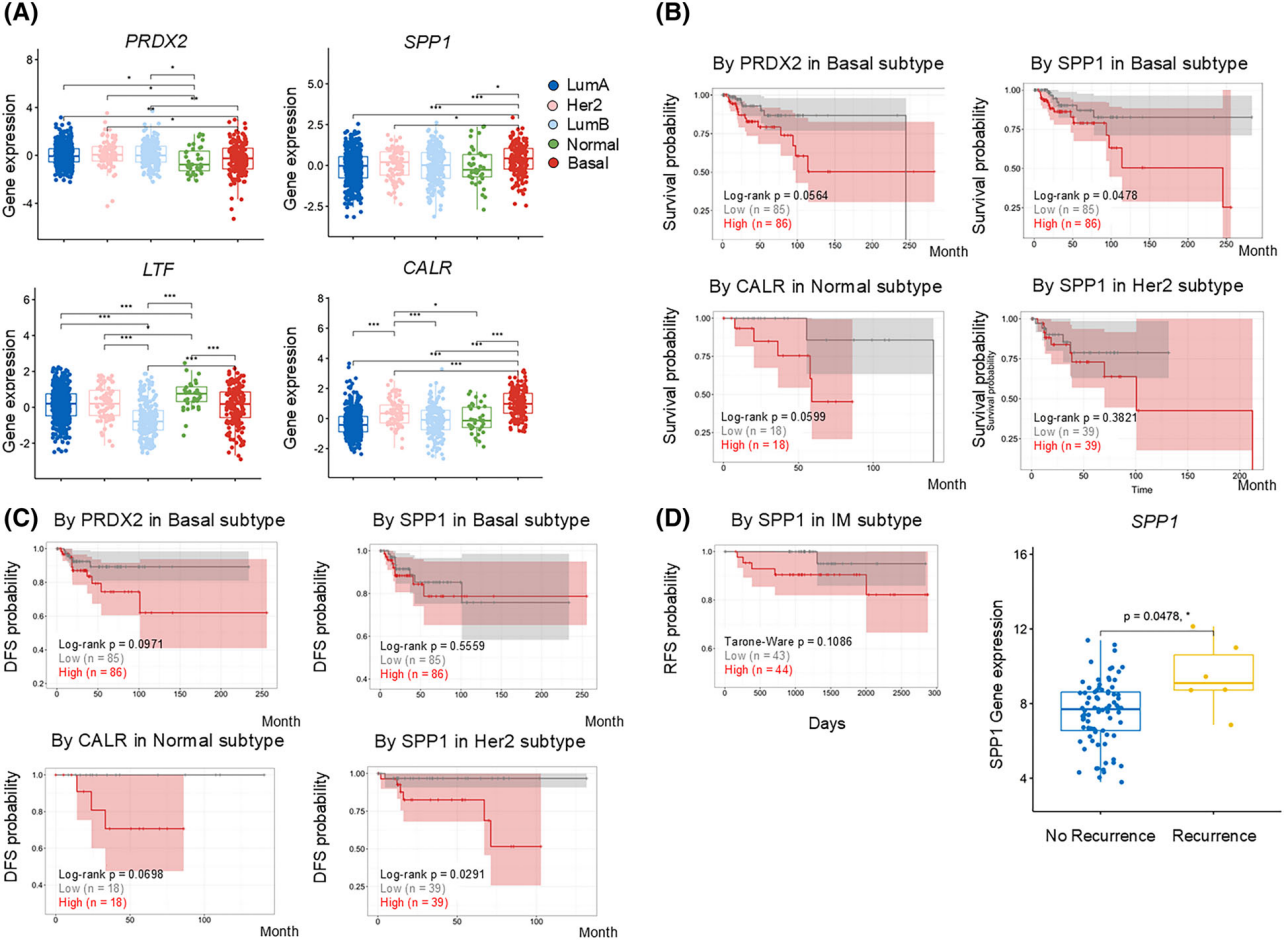
Correlations between potential biomarkers and survival rate of patients with human breast cancer. (A) Box plots representing the expression levels of specific mRNA (PRDX2, SPP1, LTF, and CALR) across different breast cancer subtypes within the TCGA BRCA dataset (Luminal A–499, Luminal B–197, Her2–78, Normal like – 36, Basal–171 patients). Comparisons were performed using Student's *t*‐test (error bars represent 25–75 percentiles, *P < 0.05, **P < 0.01, ***P < 0.001). (B) Kaplan–Meier survival plots for selected proteins (PRDX2 and SPP1) in the basal subtype, CALR in the normal subtype, and SPP1 in the HER2 subtype of breast cancer, with log‐rank *P*‐values indicated. (C) Disease‐free survival (DFS) plots for the proteins PRDX2, SPP1, and CALR across basal and other subtypes within BRCA, with log‐rank *P*‐values shown. (D) Recurrence‐free survival (RFS) plot for SPP1 in the immunomodulatory (IM) subtype from the FUSCC triple negative breast cancer (TNBC) dataset, alongside a box plot showing expression levels of SPP1 in patients with and without recurrence, indicating statistical significance (error bars represent 25–75 percentiles, *P < 0.05).

To assess whether our findings could be translated into humans, we performed a risk assessment of the four biomarkers in real‐world data using TCGA BRCA and FUSCC TNBC datasets. Within the TCGA BRCA dataset, basal‐like (TNBC group) SPP1 and CALR were elevated compared with other subtypes (Fig. 6a). Conversely, PRDX2 showed no significant difference, and LTF exhibited the highest expression in normal samples.

When analyzing overall survival, the expression levels of four biomarkers did not significantly affect the survival rates across the entire cohort. However, in the basal‐like group, which predominantly consisted of patients with TNBC, high PRDX2 and SPP1 expression levels were associated with lower survival rates (Fig. 6b). In the normal category, CALR influenced survival rates, while SPP1 showed a slight effect on survival rates in the HER2 group.

Given the relevance of SPP1 and PRDX2 in the survival rates of patients with basal‐like BRCA, we further investigated their association with DFS, focusing on recurrence or metastasis. PRDX2 was linked to recurrence in basal‐like patients, and CALR showed a similar association in the normal patient group (Fig. 6c). While SPP1 levels did not correlate with recurrence in the basal group, high SPP1 levels were significantly associated with recurrence in the HER2 patient group.

Although SPP1 did not correlate with recurrence rates in the basal patient group, suggesting no direct association, further analysis was conducted using the FUSCC TNBC dataset, which focuses exclusively on basal‐like patients. This analysis revealed that high SPP1 expression in the immunomodulatory subgroup of patients with TNBC was associated with increased recurrence rates (Fig. 6d). In 2020, Anna et al. reported that high SPP1 expression correlated with higher recurrence rates in tamoxifen‐treated patients with BRCA [24], further supporting the notion that SPP1 expression affects recurrence across diverse datasets.

## 4 Discussion

A major goal of cancer biomarker research is the development of noninvasive tests for early cancer detection. Blood is a valuable diagnostic tool because of its easy accessibility and the extensive information it carries about disease progression, as reflected by various proteins. Numerous studies have explored blood‐based biomarkers for disease diagnosis, including BRCA [25, 26, 27]. However, blood plasma‐based biomarkers associated with the systemic immunosuppressive state of patients with cancer have not been thoroughly investigated. Current proteomic technologies enable large‐scale and in‐depth investigations of proteins, particularly screening for potential biomarkers in complex biological matrices, in a high‐throughput manner [28]. In this regard, our results provide comprehensive information representing systemic responses during malignant cancer progression in a temporal manner and offer insights into the plasma biomarkers possibly involved in the host's immunosuppressive status.

Pairwise analysis of benign and malignant syngeneic models clearly explained the host response to cancer cells. Upon challenge with benign, non‐metastatic, and non‐immunosuppressive tumors, the host efficiently and continuously boosts the anticancer adaptive immune response to eradicate the altered self‐cells. In contrast, malignant and immunosuppressive cancer cells first induce systemic metabolic responses, including the carbohydrate catabolic process, in the early phase. This implies that malignant cancer cells can lead to cachexia, affecting the entire organism relatively quickly [29], although the involvement of MDSCs in inducing cachexia or vice versa remains unclear. Additionally, systemic enrichment of the leukocyte adhesion machinery would facilitate the infiltration of G‐MDSCs into pre‐metastatic organs, which accounted for over 80% of the total blood leukocytes in the case of 4T1 cells (Fig. 1d). Collectively, these phenomena aid in further acceleration of the negative feedback loop between immune suppression and metastasis.

Three (SPP1, CALR, and PRDX2) of the four enriched biomarkers in this study were found to be expressed mainly in cancer cells. This indicates that cancer cells drive systemic immune suppression, possibly by orchestrating the development of MDSCs from distant sites. Given that MDSCs recruit additional MDSCs through positive feedback mechanisms via the action of metalloproteinase‐9 [30, 31], LTF expression in MDSCs may be associated with this feedback loop [32]. Cancer‐supportive activities provide a rationale for therapeutic approaches to target cancer‐associated MDSCs, which can be achieved by understanding the mechanisms of MDSC expansion, recruitment, and immunosuppressive processes [33]. One strategy is to inhibit tumor‐ or MDSC‐derived factors that expand and mobilize MDSCs in the bone marrow or spleen. From this perspective, our results provide insights into the therapeutic modulation of systemic immunosuppression in patients with cancer.

SPP1 is a well‐established cancer biomarker [34]. As a matricellular protein, it is rapidly upregulated in response to homeostatic perturbations, contains binding sites for extracellular matrix structural proteins, and may modulate the activity of specific growth factors. Many of these extracellular matrix proteins have important functions in physiological tissue repair but are also involved in pathological conditions of tissue remodeling, such as fibrosis and tumorigenesis [35]. Our results demonstrated that SPP1 is mainly produced by cancer cells and is significantly increased in the plasma of polymorphonuclear myeloid‐derived suppressor cell (PMN‐MDSC)‐dominant tumor‐bearing mice. The functional aspect of SPP1 seems to directly affect the suppressive abilities of M‐MDSCs [36] and T cells [37] because tumor‐derived OPN supports the production in neoplastic cells of vascular endothelial growth factor and interleukin 6, which, in turn, likely sustains the expression of arginase 1 and nitric oxide synthase 2 in M‐MDSCs or T cells. Other aspects also exist; for example, CT26 colon carcinoma cells constitutively produce OPN‐induced extramedullary hematopoiesis with expansion of both PMN‐ and M‐MDSCs, whereas when silenced for OPN expression, they were blunted for this function, at least in the spleen [38]. In addition, SPP1 has been suggested as a plasma biomarker for hepatocellular carcinoma, cirrhosis [39], and BRCAs [40, 41, 42, 43]. These results strongly support the use of SPP1 as a plasma biomarker for patients with malignant and immunosuppressive cancers.

CARL, a key alleviator of endoplasmic reticulum stress, influences cancer progression, cell survival, and metastasis [44]. For example, CARL protein levels correlate with tumor growth and metastatic potential in patients with BRCA [45], and Lin et al. recently suggested that CARL could serve as a potential biomarker for invasive breast carcinoma [46]. PRDX2 has also been implicated in promoting cancer development, progression, and metastasis [47, 48]. PRDX2 is overexpressed in BRCA, and its expression levels correlate with resistance to radiation therapy in radioresistant BRCA cells [49, 50]. In our study, PRDX2 expression significantly increased in plasma at an early stage in 4T1‐bearing mice. Given that PRDX2 is associated with lower survival rates in patients with TNBC and cancer recurrence, PRDX2 could be a meaningful biomarker for the early detection of MDSC‐dominant BRCA. However, the correlation between PRDX2, CARL, and MDSC generation has not yet been studied here, and further studies are required.

A limitation of our study is the lack of statistical power, which may increase the likelihood of both Type I and Type II errors. This arises from the relatively small sample sizes, with only three biological replicates, and the inherent complexity of cancer‐related systemic responses. While the observed trends in plasma proteomic changes and the proposed biomarkers provide valuable insights, they should be interpreted cautiously. Future studies involving larger sample sizes and rigorous experimental designs are essential to validate these findings and strengthen their reliability.

In conclusion, plasma proteomic analysis of 4T1‐ and 67NR‐bearing mice highlights that the changes in the plasma proteome that occur during cancer development arise from various sources. These include nonspecific systemic reactions involving acute‐phase proteins such as those produced by the liver; other host responses that are more specific to BRCA but not directly tumor‐derived, such as proteins involved in immune cell recruitment, signaling, and metabolic pathways; and proteins originating directly from the tumor and local microenvironment. The differences observed in plasma proteins between the 4T1 and 67NR models, particularly the proteins specifically increased in 4T1, may be associated with the generation of host immunosuppressive status. In this context, we propose four potential biomarkers, SPP1, LTF, CARL, and PRDX2, for the early detection of MDSC‐dominant cancer.

## 5 Conclusions

We collected plasma samples from naive and syngeneic breast tumor mouse models, including benign 67NR and malignant 4T1 models, and subjected these samples to LC–MS analysis to identify changes in the plasma proteome associated with immunosuppressive cancer and potential biomarkers for predicting systemic immunosuppression. Among the 27 proteins exhibiting group‐specific expression in the blood of 4T1 models, immune‐related proteins such as OPN, LTF, CALR, and PRDX2 were identified as potential biomarkers of MDSC‐producing breast cancer. These biomarkers were expressed in either cancer cells or MDSCs within the 4T1 model. Additionally, osteopontin and peroxiredoxin 2 were correlated with low survival probability and high recurrence rates in patients with TNBC. Our findings suggest that MDSC‐producing immunosuppressive cancers possess unique plasma proteomes, providing additional insights into the immune status of cancer.

## Conflict of interest

The authors declare no competing interests.

## Author contributions

YRN, KPK, and SHS conceptualized, primarily investigated, and acquired the funds for this study. YJP, JWO, and YRN wrote the original manuscript and designed the figures. JWO, HWC, JWK, and YRN performed the experiments and analyzed the data. YJP interpreted the data and reviewed/revised the manuscript.

## Peer review

The peer review history for this article is available at https://www.webofscience.com/api/gateway/wos/peer‐review/10.1002/1878‐0261.13817.

## Supporting information


**Table S1.** Identified proteins.

**Table S2.** Quantifiable proteins.

## Data Availability

Supplemental data are available online, and the datasets used in this study can be obtained from the corresponding author upon request.
